# Structural and Optical Sensing Properties of Nonthermal Atmospheric Plasma-Synthesized Polyethylene Glycol-Functionalized Gold Nanoparticles

**DOI:** 10.3390/nano11071678

**Published:** 2021-06-25

**Authors:** Linh Nhat Nguyen, Pradeep Lamichhane, Eun Ha Choi, Geon Joon Lee

**Affiliations:** 1 Department of Electrical and Biological Physics, Kwangwoon University, Seoul 01897, Korea; nhatlinhusth@gmail.com (L.N.N.); theprodip@gmail.com (P.L.); ehchoi@kw.ac.kr (E.H.C.); 2 Plasma Bioscience Research Center, Kwangwoon University, Seoul 01897, Korea; 3 Laboratory of Plasma Technology, Institute of Materials Science, Vietnam Academy of Science and Technology, 18 Hoang Quoc Viet, Hanoi 100000, Vietnam

**Keywords:** gold nanoparticles, plasma synthesis, polyethylene glycol, reactive oxygen species, surface plasmon resonance, surface-enhanced Raman scattering, optical sensing

## Abstract

Polyethylene glycol-functionalized gold nanoparticles (Au@PEG NPs) were prepared by a simple plasma-assisted method without additional reducing chemicals. After irradiating tetrachloroauric acid (HAuCl_4_) and polyethylene glycol (PEG) in aqueous medium with an argon plasma jet, the gold precursor transformed into an Au@PEG NP colloid that exhibited surface plasma resonance at 530 nm. When the plasma jet entered the water, additional reactive species were induced through interactions between plasma-generated reactive species and aqueous media. Interaction of the gold precursor with the plasma-activated medium allowed the synthesis of gold nanoparticles (AuNPs) without reductants. The plasma-synthesized Au@PEG NPs had a quasi-spherical shape with an average particle diameter of 32.5 nm. The addition of PEG not only helped to stabilize the AuNPs but also increased the number of AuNPs. Au@PEG NP-loaded paper (AuNP-paper) was able to detect the degradation of rhodamine B, therefore, indicating that AuNP-paper can act as a surface-enhanced Raman scattering platform. Dye degradation by plasma treatment was investigated by optical absorption and Raman spectroscopy. The method proposed for the fabrication of Au@PEG NPs is rapid, low-cost, and environment-friendly and will facilitate the application of plasma-synthesized nanomaterials in sensors.

## 1. Introduction

Metal nanoparticles are used in various applications, such as optical sensing, bioimaging, drug delivery, and photothermal therapy. Metal nanoparticle-based optical sensing is a noninvasive method that allows for the rapid diagnosis of biomaterials and cancers [1]. Owing to their outstanding physical and optical properties, gold nanoparticles (AuNPs) have become one of the most prominent nanomaterials in the nanotechnology field. AuNPs are used widely as surface-enhanced-Raman spectroscopy (SERS) platforms due to optical confinement effects stemming from their surface plasmon resonance [2,3]. Generally, AuNPs can be synthesized by chemical reduction of ${\mathrm{HAuCl}}_{4}$ precursors. However, harsh reducing agents are used during chemical synthesis, and AuNP toxicity has become a serious concern [4]. To overcome this issue, the development of green synthesis processes that eliminate the use of toxic reagents during the preparation of AuNPs has become an important research direction [5,6]. Using green synthesis, environmentally and biologically friendly AuNPs suitable for a wide range of applications, including optical sensing, biomedical imaging, and physicochemical analysis, can be prepared. In this context, many studies have reported green preparations of gold nanoparticles using plant extracts, microorganisms, enzymes, polysaccharides, and other non-toxic molecules [7,8,9,10,11]. These substances contain large amounts of hydroxyl and phenol groups that reduce Au ions [12]. Generally, however, these non-hazardous substances are weak reducing agents, thus, resulting in longer processing times compared with that of chemical synthesis [13]. In this sense, non-thermal plasma is a beneficial alternative to synthesize AuNPs, because toxic agents are not used, and the processing time is shorter than that required for chemical synthesis. 

The non-thermal plasma synthesis of metal nanostructures is an alternative green synthesis approach that has substantial advantages compared to chemical synthesis [14,15,16,17]. This method uses non-thermal atmospheric pressure plasmas under ambient conditions and produces large amounts of reactive oxygen and nitrogen species, excited metastable species, free electrons, and charged particles [18]. These species can be transferred into the liquid phase when the plasma is in contact with liquid medium [19,20]. Dissolved reactive species are key contributors to the chemical reactions that occur during the plasma synthesis of metallic nanoparticles in the plasma-activated water [21,22]. In the plasma synthesis of AuNPs, the reduction of ${\mathrm{Au}}^{3+}$ ions can be triggered both by short-lived solvated electrons and long-lived reactive species, such as $H_{2}O_{2}$, and reduction of the gold precursor results in surfactant-free AuNPs [23]. Plasma synthesis of hybrid nanomaterials composed of AuNPs and a polymer has been investigated to improve the stability and biocompatibility of AuNPs [24,25,26]. Additional polymers play the role of stabilization agents that can assist the formation of AuNPs in the plasma-treated medium. The amount of plasma-synthesized AuNPs has been reported to be higher in ${\mathrm{HAuCl}}_{4}–$polymer than in ${\mathrm{HAuCl}}_{4}$ [25]. To improve the polyethylene glycol (PEG) bonding strength to the AuNP surface, it is necessary to modify PEG with thiol or amine groups [27]. For instance, Furusho et al. functionalized PEG with pentaethylenehexamine (PEHA) and used it as a polymer matrix for plasma synthesis of AuNPs [28]. PEG is a biocompatible polymer that has been extensively used for nanoparticle stabilization and surface modification [29]. There are numerous reports describing the preparation and applications of PEG-coated plasmonic nanomaterials, including optical sensing and biomedical applications [30,31].

**Figure 1:**
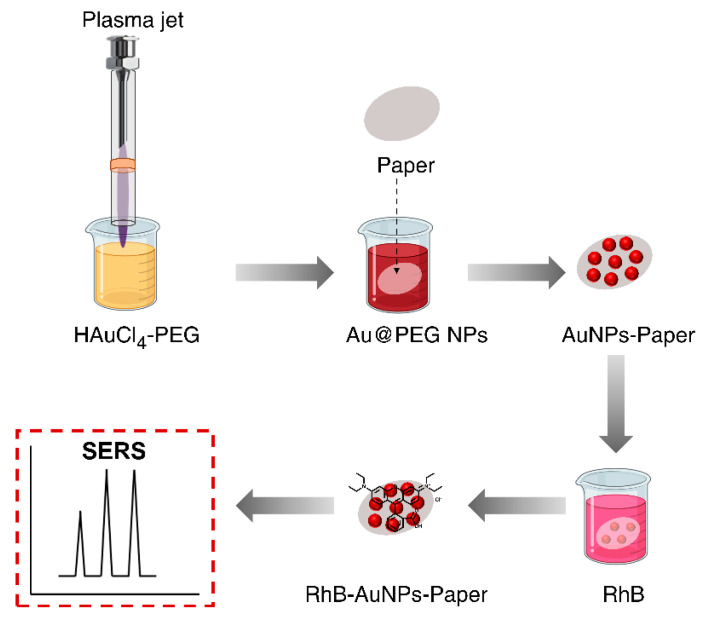
Schematic representation of the plasma-synthesis of polyethylene glycol-functionalized gold nanoparticles (Au@PEG NPs) and fabrication of a surface-enhanced Raman scattering platform from Au@PEG NPs.

In this study, we describe the synthesis of gold nanoparticles by plasma reduction of a ${\mathrm{HAuCl}}_{4}$ precursor. The morphological and optical properties of AuNPs were examined by scanning electron microscopy (SEM) images and optical absorption spectra, respectively. PEG was used to improve the morphology and formation efficiency of nanoparticles during plasma synthesis of AuNPs. Next, we investigated the optical sensing properties of AuNPs using plasma-treated and untreated rhodamine B (RhB) dye. The degradation of RhB by plasma treatment was studied by optical absorption and Raman spectroscopy. For Raman spectroscopic measurements of plasma-treated RhB, AuNP-loaded paper was used as a SERS platform (Figure 1).

## 2. Experimental Details

### 2.1. Plasma Jet System

**Figure 2:**
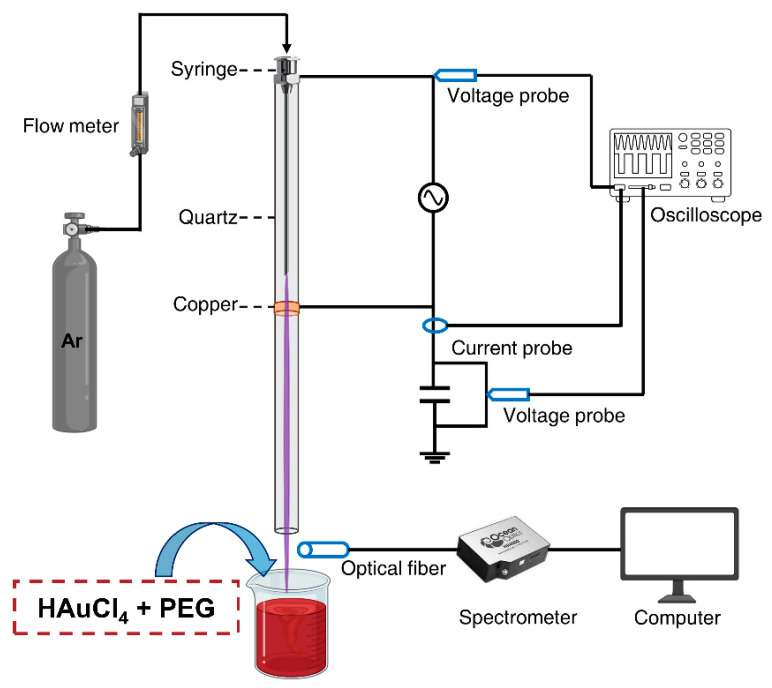
Schematic of the experimental setup for Au@PEG NP synthesis using a non-thermal atmospheric plasma jet.

Figure 2 illustrates the experimental layout of the plasma jet used to synthesize the Au@PEG NPs in this work. The plasma jet was constructed by inserting a 12-cm-long stainless-steel syringe inside a 20-cm-long quartz tube. A copper piece was wrapped around the outside of the quartz tube and connected to the ground. A high-frequency AC sinusoidal voltage of 5 kV was applied between the steel syringe and the copper piece, and 99.999% argon at a flow rate of 700 sccm was used as a feeding gas to generate a plasma jet. Current and voltage waveforms were measured using a current probe (Tektronix, P6022, Beaverton, OR, USA) and a voltage probe (Tektronix, P6015A, Beaverton, OR, USA), respectively, both connected to a digital oscilloscope (Lecroy, WaveSurfer 434, New York, NY, USA). Optical emission spectra (OES) of the plasma plume were recorded by a high-resolution fiber optic spectrometer (Ocean Optics, HR4000, Orlando, FL, USA).

### 2.2. Synthesis of Gold Nanoparticles by Plasma Jet

Tetrachloroauric acid trihydrate (${\mathrm{HAuCl}}_{4}\cdot 3H_{2}O$, ≥99.9% trace metals basis) and polyethylene glycol (PEG, $M_{n}=4000$) were purchased from Sigma Aldrich (Yongin-city, Kyunggi-do, Korea) and used without purification. All glassware was cleaned with aqua regia solution (a 3:1 mixture of hydrochloric acid and nitric acid) overnight and washed thoroughly with de-ionized (DI) water before use. Stock solutions of 10 mM ${\mathrm{HAuCl}}_{4}$ and 50 mM PEG were prepared. A mixture of ${\mathrm{HAuCl}}_{4}$ and PEG was prepared by adding 300 μL of 10 mM ${\mathrm{HAuCl}}_{4}$ and 50 μL of 50 mM PEG to 4 mL of DI water. The ${\mathrm{HAuCl}}_{4}–\mathrm{PEG}$ solution was treated with the non-thermal plasma jet for 10 min with vigorous magnetic stirring, which resulted in a dark purple Au@PEG solution. The obtained product was purified by centrifugation at 8000 rpm for 10 min, followed by removal of the supernatant.

### 2.3. Morphological and Optical Characterizations of Plasma-Synthesized Gold Nanoparticles

The SEM analysis of plasma-synthesized Au@PEG NPs was performed by a field-emission SEM (JEOL, JSM–7001F, Tokyo, Japan) under 15 kV acceleration voltage. For better SEM observation, we functionalized Au@PEG NPs with bovine serum albumin (BSA) to prevent the aggregation of gold nanoparticles [32]. Briefly, 100 mL of Au@PEG NP solution was mixed with 100 mL of 300 μM BSA. Ten microliters of the Au@PEG NP–BSA mixture was drop-cast onto a silicon wafer and dried under vacuum conditions. The absorption spectra of plasma-synthesized Au@PEG NP solutions were measured using an optical absorption spectrometer (Jasco, J–815, Tokyo, Japan).

### 2.4. Coating of Gold Nanoparticles on Filter Paper

Filter paper (Hyundai Micro, HM5301200, Seoul, Korea) was cut into 1.0-cm-diameter disk-shaped pieces and dipped in Au@PEG NP solution for 12 h at 4 °C. After dip-coating, the Au@PEG NP-loaded paper (AuNP-paper) was dried under ambient conditions.

### 2.5. Optical Properties of Rhodamine B before and after Plasma Treatment

Rhodamine B (RhB, analytical standard) was purchased from Sigma Aldrich. A $10^{-4}\;M$ RhB stock solution was prepared by dissolving 4.8 mg of RhB powder in 100 mL DI water, and this solution was filtered to eliminate insoluble components. A 3-mL aliquot of $10^{-4}\;M$ RhB solution was treated with an Ar plasma jet for different amounts of times (0, 90, 180, and 600 s). The optical absorption spectra of plasma-treated and untreated RhB solutions were obtained by an optical absorption spectrometer (Jasco, J–815, Tokyo, Japan). A paper disk plate was dip-coated in the plasma-treated RhB solution for 12 h and dried at room temperature. Raman spectra of plasma-treated and untreated RhB on the AuNP-paper were measured at room temperature using a confocal Raman microscope (WITec, Alpha 300R, Ulm, Germany) with a 633-nm excitation light emitted from a He-Ne laser. In order to obtain the Raman spectra, the laser beam was focused on the sample using a microscope objective (20×).

## 3. Results and Discussion

### 3.1. Plasma Discharge Characterization

**Figure 3:**
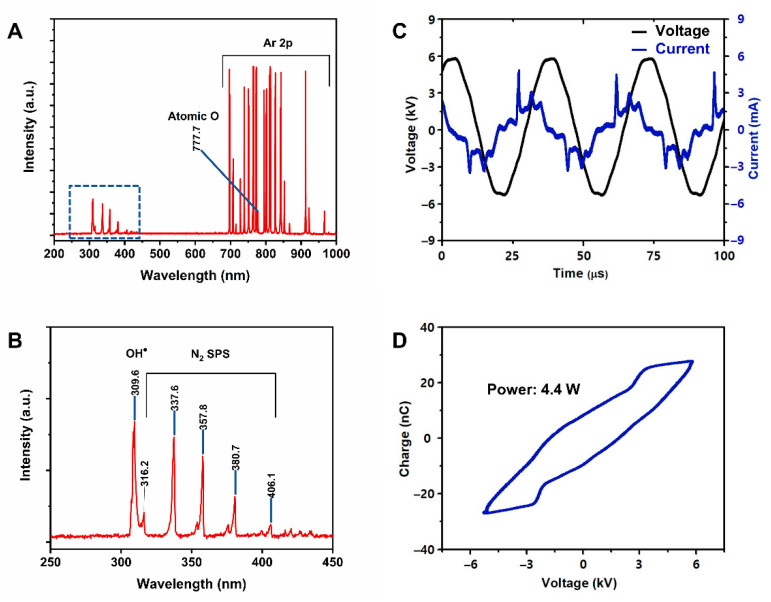
Characteristics of the plasma jet. (**A**,**B**) Optical emission spectrum, (**C**) current-voltage waveform, and (**D**) Lissajous curve of the Ar plasma jet.

OES can provide important information about reactive species contained in a plasma jet. Figure 3A demonstrates the OES of the plasma jet over the wavelength range of 200–1000 nm; strong emission lines indicate the presence of plasma-generated reactive species. The 309-nm emission of the hydroxyl radical (OH) originated from dissociation of water vapor in ambient air [33]. Weak emission lines of the nitrogen second positive system ($N_{2}$–SPS; $C^{3}\Pi_{u}\to B^{3}\Pi_{g}$ transition of nitrogen molecules) were detected in the range of 330–380 nm [34] (Figure 3B), which is attributed to the fact that the plasma was operated under atmospheric conditions. Strong emission lines in the wavelength range of 700–1000 nm were attributed to the Ar 2p→1s transition, because argon was used as the working gas. These generated species can penetrate further into the liquid medium under the effect of gas flow and contribute to plasma–liquid interactions. Figure 3C shows the current-voltage profile of the AC-driven plasma discharge. The discharge current peaks appeared in the positive half-cycle of the applied voltage are due to the accumulation of positive surface charges while the polarity of these charges is reversed in the negative half-cycle of the applied voltage. 

The measured peak voltage and peak current were about 6 kV and 5 mA, respectively. The period of voltage waveform was 35 µs, which corresponds to a frequency of 28.5 kHz. We connected a 33-nF ceramic capacitor in series with the ground electrode and measured the voltage across this capacitor to calculate the accumulated charge. A charge-voltage Lissajous loop was constructed by plotting the accumulated charge versus the applied voltage (Figure 3D). The dissipated power of the plasma jet was calculated to be 4.4 W based on the area of the Lissajous loop.

### 3.2. Characteristics of Plasma-Treated Water for Plasma Synthesis of Gold Nanoparticles

**Figure 4:**
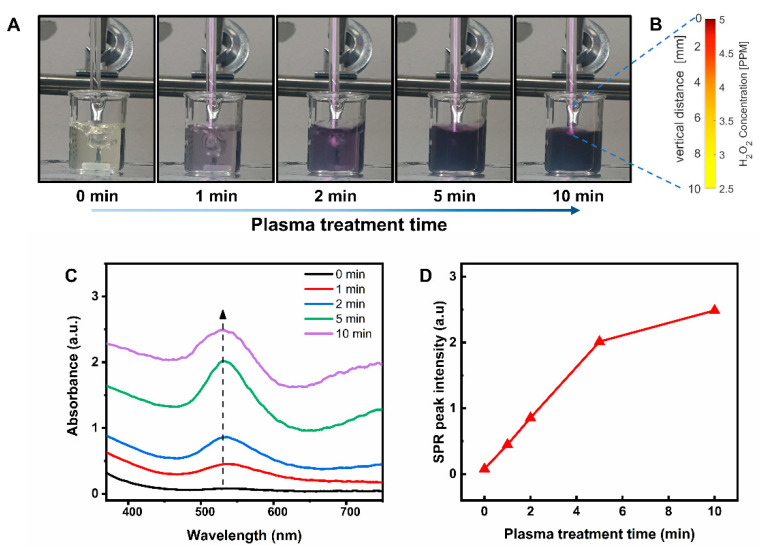
Effects of the plasma treatment time on the optical properties of the Au@PEG NP solution produced by plasma treatment of a ${\mathrm{HAuCl}}_{4}$−PEG solution. (**A**) The color change of the Au@PEG NP solution over a plasma treatment time of 10 min. (**B**) Spatial distribution of the $H_{2}O_{2}$ concentration along the plasma jet. (**C**) Optical absorption spectra of the AuNP solutions with different plasma treatment times. (**D**) SPR peak intensity of the AuNP solution as a function of the plasma treatment time.

When a plasma jet enters water, reactive species in the plasma phase are transported to the liquid phase. These solvated species significantly change the physicochemical properties of plasma-treated water and stimulate a series of complex chemical reactions. Previous studies have confirmed that hydrogen peroxide ($H_{2}O_{2}$) is a key factor in the plasma reduction process of ${\mathrm{Au}}^{3+}$ [23,25]. $H_{2}O_{2}$ is a long-lived species that is generated through several pathways [35,36,37]. In the plasma jet, gaseous $H_{2}O_{2}$ formed inside the quartz tube is delivered through the nozzle, and aqueous $H_{2}O_{2}$ is generated continuously at the gas−liquid interface and accumulated inside the bulk liquid, resulting in a high concentration of $H_{2}O_{2}$. In the gas phase, plasma-generated OH radicals can recombine to form gaseous $H_{2}O_{2}$, which can be transported to the gas–liquid interface (Equation (1)) [35]. $H_{2}O_{2}$ was observed at all positions along the plasma jet, as shown in Figure 4B. Most of the $H_{2}O_{2}$ formed in the plasma phase is immediately dissolved in water. At the gas–liquid interface, plasma-generated OH radicals can be converted to aqueous $H_{2}O_{2}$, which can be transported into the liquid medium (Equation (2)) [35]. In the bulk liquid, OH radicals can be generated through plasma-induced ultraviolet (UV) photolysis of water, and solvated OH radicals can recombine to form $H_{2}O_{2}$ (Equation (3)) [35]. Under acidic conditions, plasma-generated superoxide can be converted to $H_{2}O_{2}$ (Equation (4)) [36].

(1) $${\mathrm{OH}}_{\left(g\right)}+{\mathrm{OH}}_{\left(g\right)}\to H_{2}O_{2}{}_{\left(g\right)}$$

(2) $${\mathrm{OH}}_{\left(g\right)}+{\mathrm{OH}}_{\left(g\right)}\to {\mathrm{OH}}_{\left(\mathrm{aq}\right)}+{\mathrm{OH}}_{\left(\mathrm{aq}\right)}\to H_{2}O_{2}{}_{\left(\mathrm{aq}\right)}$$

(3) $$H_{2}O_{\left(\mathrm{aq}\right)}\overset{\mathrm{UV}}{\to }H_{\left(\mathrm{aq}\right)}+{\mathrm{OH}}_{\left(\mathrm{aq}\right)};\;{\mathrm{OH}}_{\left(\mathrm{aq}\right)}+{\mathrm{OH}}_{\left(\mathrm{aq}\right)}\to H_{2}O_{2}{}_{\left(\mathrm{aq}\right)}$$

(4) $$O_{2}^{-}{}_{\left(\mathrm{aq}\right)}+{\mathrm{HO}}_{2}{}_{\left(\mathrm{aq}\right)}+H^{+}{}_{\left(\mathrm{aq}\right)}\to H_{2}O_{2}{}_{\left(\mathrm{aq}\right)}+O_{2}{}_{\left(\mathrm{aq}\right)}$$

Consequently, each of these four equations represents possible pathways to generate $H_{2}O_{2}$ by an atmospheric plasma jet; (1) $H_{2}O_{2}$ in gas phase, (2) $H_{2}O_{2}$ at gas–liquid interface, (3) $H_{2}O_{2}$ via UV photolysis of water, and (4) $H_{2}O_{2}$ via plasma-generated superoxide.

In the plasma-activated water, aqueous $H_{2}O_{2}$ can be produced by Equations (2)–(4) [35,36,37]. To verify the generation of $H_{2}O_{2}$ by the plasma jet, we treated 4 mL of DI water with a plasma jet for 10 min and quantified the $H_{2}O_{2}$ concentration of the plasma-treated water. By assessing the absorption spectrum of the plasma-treated water using a standard $H_{2}O_{2}$ detection colormetric assay, we determined the concentration of $H_{2}O_{2}$ to be about 3.3 mM. $H_{2}O_{2}$ concentration in the plasma-activated water is relatively high due to the accumulation of aqueous $H_{2}O_{2}$ generated at the gas–liquid interface and inside the bulk liquid [35,36,37].

### 3.3. Optical and Morphological Properties of Plasma-Synthesized Gold Nanoparticles

The prepared ${\mathrm{HAuCl}}_{4}–\mathrm{PEG}$ solution was stable at room temperature for several hours with no significant change in color, suggesting no reduction of ${\mathrm{Au}}^{3+}$ ions. After the start of plasma treatment, the color of the ${\mathrm{HAuCl}}_{4}–\mathrm{PEG}$ solution rapidly changed from light yellow to dark purple, indicating the formation of AuNPs (Figure 4A). The concentration of gold nanoparticles in solution was proportional to the absorption coefficient of the surface plasmon resonance (SPR). Therefore, the absorption spectra of ${\mathrm{HAuCl}}_{4}–\mathrm{PEG}$ solutions with different plasma treatment times were measured to evaluate the nanoparticle formation (Figure 4C). The observed increase in the SPR peak intensity with the plasma treatment time is summarized in Figure 4D. These results demonstrate that plasma treatment was an effective method to synthesize Au@PEG NPs. The plasma reduction mechanism of ${\mathrm{Au}}^{3+}$ to metallic ${\mathrm{Au}}^{0}$ has been well-established and is based on the following two reactions:

(5) $${\mathrm{Au}}^{3+}+3e^{-}\to {\mathrm{Au}}^{0}$$

(6) $$2{\mathrm{Au}}^{3+}+3H_{2}O_{2}\to 2{\mathrm{Au}}^{0}+3O_{2}+6H^{+}$$

Equation (5) is a direct reduction of ${\mathrm{Au}}^{3+}$ to ${\mathrm{Au}}^{0}$ by plasma electrons solvated in the liquid medium [38]. Equation (6) shows an indirect reduction of ${\mathrm{Au}}^{3+}$ through $H_{2}O_{2}$ that is formed in the medium due to plasma treatment [23]. As $H_{2}O_{2}$ is a stable species with a long lifetime, Equation (6) can proceed after the plasma is turned off. We also treated a ${\mathrm{HAuCl}}_{4}$ solution with plasma without adding PEG. The color of the plasma-treated ${\mathrm{HAuCl}}_{4}$ solution changed slightly to light orange, thereby, suggesting that the reaction occurred slower.

**Figure 5:**
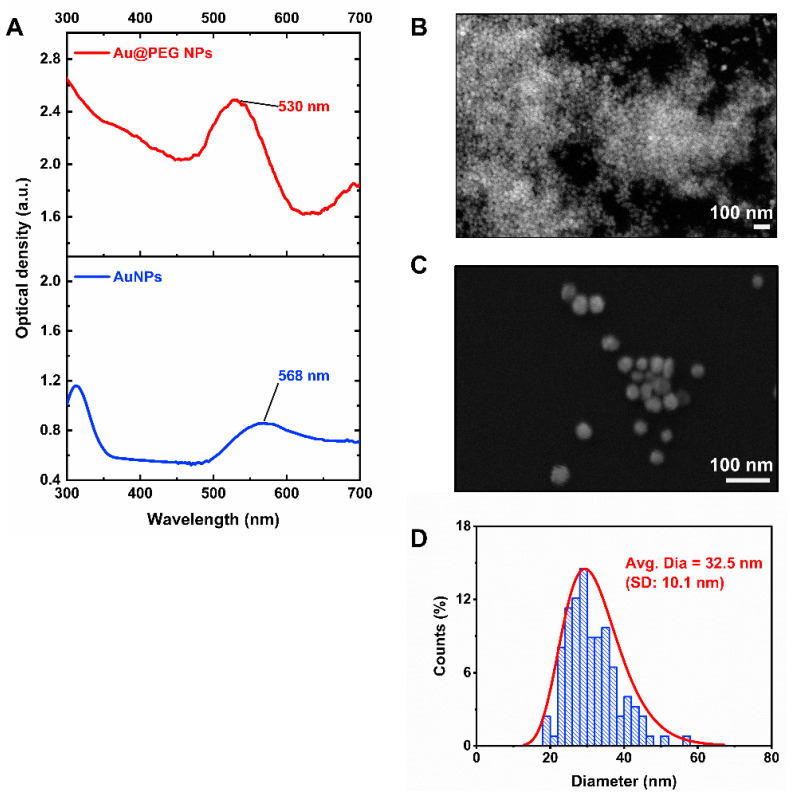
(**A**) Optical absorption spectra of the Au@PEG NP solution after plasma treatment for 10 min: ${\mathrm{HAuCl}}_{4}–\mathrm{PEG}$ (red curve) and ${\mathrm{HAuCl}}_{4}$ (blue curve). (**B**) 60,000× and (**C**) 160,000× SEM images of Au@PEG NPs. (**D**) The particle size distribution of Au@PEG NPs. The average particle size was 32.5 nm with a standard deviation of 10.1 nm.

Figure 5A shows the optical absorption spectra of the AuNP solutions produced by plasma treatment of the ${\mathrm{HAuCl}}_{4}–\mathrm{PEG}$ and ${\mathrm{HAuCl}}_{4}$ solutions. The plasma-treated ${\mathrm{HAuCl}}_{4}–\mathrm{PEG}$ solution exhibited an absorption peak at 530 nm, while the plasma-treated ${\mathrm{HAuCl}}_{4}$ solution had an absorption peak at 567 nm. These results suggest that the average nanoparticle size of the plasma-treated ${\mathrm{HAuCl}}_{4}$ solution was larger than when PEG was present. Small-sized ${\mathrm{Au}}^{0}$ seeds formed rapidly under plasma treatment and grew continuously, resulting in larger AuNPs. When there was no surfactant in the solution, AuNPs were free to grow in size during plasma treatment, resulting in larger AuNPs. When PEG was added, it functioned as a surfactant to stabilize AuNPs, thus, limiting their growth. The SPR peak intensity of plasma-treated ${\mathrm{HAuCl}}_{4}–\mathrm{PEG}$ solution (optical density of 2.5) was much stronger than that of the plasma-treated ${\mathrm{HAuCl}}_{4}$ solution (optical density of 0.8), indicating the formation of more AuNPs in the former solution. PEG likely functioned as a stabilizer and promoted the formation of AuNPs. This result is in agreement with a previous report of De Vos et al., who performed plasma synthesis of AuNPs with an additional polymer matrix [25]. They demonstrated that the amount of plasma-synthesized AuNPs was higher in ${\mathrm{HAuCl}}_{4}–\mathrm{PEG}$ solution than in ${\mathrm{HAuCl}}_{4}$ only solution. Based on the obtained data, the addition of PEG to the plasma-synthesis process of AuNPs affected not only the size of the resultant AuNPs but also the number of AuNPs. 

The morphology of Au@PEG NPs was studied by SEM analysis. Figure 5B,C shows 60,000× and 160,000× magnification SEM images of Au@PEG NPs loaded on a silicon substrate, respectively. Plasma-synthesized Au@PEG NPs with a quasi-spherical shape were observed. The particle size distribution of Au@PEG NPs was obtained from SEM images using *ImageJ* software, v1.52a; National Institutes of Health, MD, USA, 2018 [39]. The average particle diameter of Au@PEG NPs was calculated as 32.5 ± 10.1 nm (Figure 5D). We noted that there was a small fraction of Au@PEG NPs with large non-spherical shapes (triangular and hexagonal), which were usually found in plasma-assisted nanoparticle synthesis. During the plasma treatment process, the ionic gold precursor concentration continuously depleted, while the number of formed metallic gold nanoparticles increased. When the precursor concentration is low, the anisotropic growth of the formed gold nanoparticles is favored over the nucleation of new nanoparticles [23,25]. 

### 3.4. Characteristics of Gold Nanoparticles Loaded on the Paper Disk

**Figure 6:**
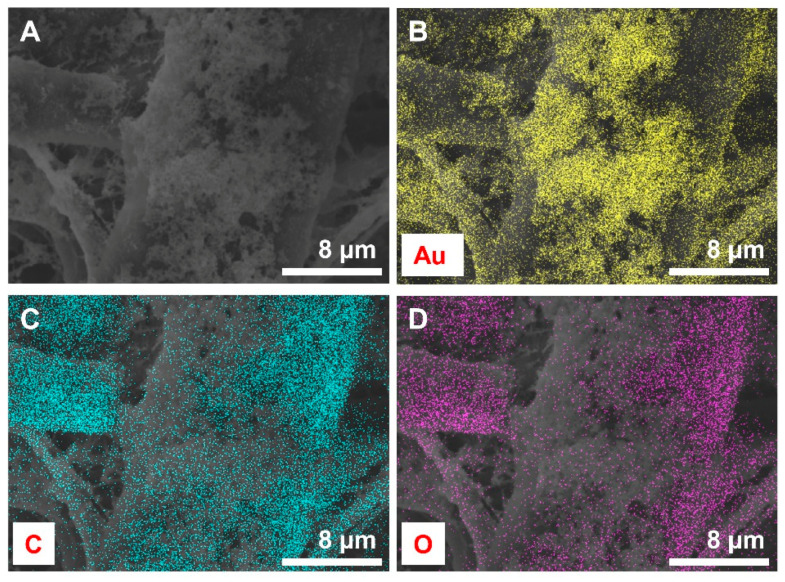
(**A**) 2300× SEM image of AuNP-paper and (**B**–**D**) the corresponding energy-dispersive X-ray spectroscopy elemental mapping, (**B**): Gold, (**C**): Carbon, and (**D**): Oxygen.

To investigate the optical sensing properties of AuNPs as a SERS platform, we deposited Au@PEG NPs on paper by dipping paper disks in Au@PEG NP colloids for 12 h. After dip-coating, the paper color changed from white to black, implying the successful deposition of Au@PEG NPs. A SEM image of AuNPs loaded on the paper is shown in Figure 6A. A large amount of Au@PEG NPs accumulated on the paper. We also performed energy-dispersive X-ray spectroscopy elemental mapping to confirm the existence of Au@PEG NPs on the paper (Figure 6B–D). Intense gold elemental signals (yellow dots) were detected across the surface of the AuNP-paper, indicating the homogenous deposition of Au@PEG NPs by the dip-coating method. O and C element signals from the paper disk plate were also present.

### 3.5. Optical Sensing Properties of Plasma-Treated Rhodamine B by Gold Nanoparticles

**Figure 7:**
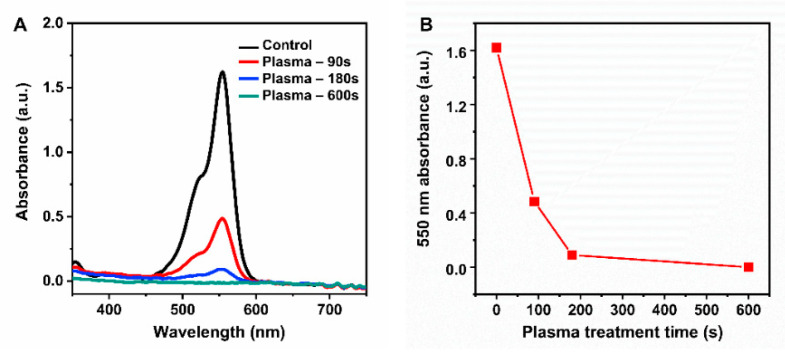
(**A**) Optical absorption spectra of the rhodamine B solutions with different plasma treatment times. The control sample corresponds to a $10^{-4}\;M$ Rh B solution not exposed to plasma. (**B**) 550 nm-absorption peak intensity of the RhB solution as a function of the plasma treatment time.

To study the optical sensing activity of plasma-synthesized gold nanoparticles, we investigated the effect of plasma treatment on rhodamine B (RhB). RhB was selected as a model analyte as it is a nonbiodegradable and commonly used organic dye. The RhB solution ($10^{-4}\;M$) was treated with a plasma jet for 0, 90, 180, and 600 s. The plasma-induced degradation of organic dyes in aqueous solution has been studied previously with optical absorption spectroscopy [40,41,42]. In this research, we investigated plasma-induced dye degradation by both optical absorption and Raman spectroscopy. Figure 7A shows the optical absorption spectra of plasma-treated and untreated RhB solutions. The pristine RhB solution exhibited a broad absorption band with a strong peak at 550 nm and a shoulder at 520 nm. The 550 nm absorption was assigned to RhB monomers, and the 520 nm absorption peak was assigned to RhB dimers [43]. The main absorption peak located at 550 nm was chosen as an indicator for the degree of dye degradation. A change in the primary absorption peak at 550 nm with increasing plasma treatment time is shown in Figure 7B. When comparing the 550 nm absorption peak of plasma-treated RhB with that of untreated RhB, the 550 nm absorption peak intensity of 90 s plasma-treated RhB was around 26% of that of pristine RhB, indicating that the RhB molecules were degraded significantly by the plasma jet. When the plasma treatment time was increased to 180 s, the absorbance of RhB was reduced to about 5% of that of pristine RhB. Plasma treatment for 600 s reduced the absorbance of RhB to almost zero, suggesting that most of the RhB molecules had been destroyed by the plasma jet.

**Figure 8:**
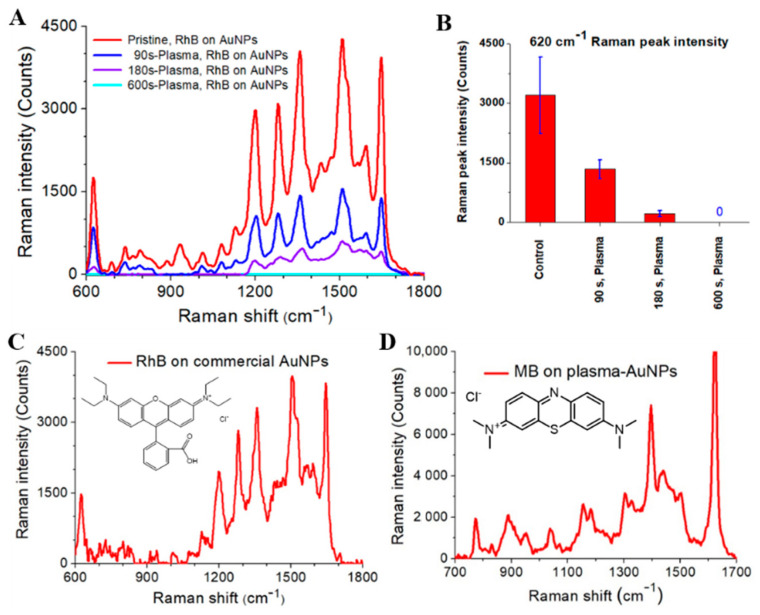
(**A**) Raman spectra of plasma-treated and untreated rhodamine B adsorbed on the AuNP-paper. The control sample was prepared using $10^{-4}\;M$ Rh B solution not exposed to plasma. The Raman spectra were averaged after measurements at five different positions on the AuNP-paper. (**B**) Effects of the plasma treatment time on the $620\;{\mathrm{cm}}^{-1}$−Raman peak. Error bars indicate the standard deviation of the Raman scattering intensities for five different positions on the AuNP-paper. (**C**) Raman spectra of the rhodamine B adsorbed on the AuNP-paper with commercial gold nanoparticles. (**D**) Raman spectra of the methylene blue adsorbed on the AuNP-paper with plasma-synthesized gold nanoparticles.

To further investigate the degradation of RhB by plasma treatment, we measured the Raman spectra of plasma-treated and untreated RhB. For SERS detection of RhB adsorbed on the AuNP-paper, paper disks were submerged in plasma-treated and untreated RhB solutions for 12 h, and RhB coated onto the AuNP-paper was subjected to Raman measurements after washing with fresh DI water. Figure 8A shows the Raman spectra of plasma-treated and untreated RhB adsorbed on the AuNP-paper. In this experiment, the Raman spectra were obtained using 633-nm excitation light with a power of 0.5 mW. The Raman spectra were measured at five different positions on the AuNP-paper. Spatially averaged Raman spectra were plotted in Figure 8A. The Raman spectra of pristine RhB exhibited Raman bands at $620.5{\;\mathrm{cm}}^{-1}$, $1197.7{\;\mathrm{cm}}^{-1}$, $1278.6{\;\mathrm{cm}}^{-1}$, $1356.6{\;\mathrm{cm}}^{-1}$, $1502.8{\;\mathrm{cm}}^{-1}$, and $1642.4{\;\mathrm{cm}}^{-1}$, which we attributed to C−C stretching, C=C stretching, and C−H bending modes of the aromatic ring [44]. The effects of the plasma treatment time on the $620{\;\mathrm{cm}}^{-1}$−Raman peak are shown in Figure 8B. Error bars indicate standard deviations of Raman scattering intensities for those five positions on the AuNP-paper. The Raman peak intensities of the major Raman bands of plasma-treated RhB decreased with increasing plasma treatment time, indicating that RhB molecules were degraded by plasma treatment.

To compare the optical sensing activity of plasma-synthesized AuNPs with that of commercial gold nanoparticles, we measured the Raman spectra of RhB coated on the AuNP-paper with commercial gold nanoparticles (Sigma-Aldrich, 741973, Yongin-city, Kyunggi-do, Korea; 30 nm diameter, OD 1, stabilized suspension in citrate buffer). Commercial AuNP-loaded papers and plasma-synthesized Au@PEG NP-loaded papers were prepared using gold nanoparticle solutions with the same SPR absorption peak. As shown in Figure 8C, the Raman spectra of RhB on commercial gold nanoparticles exhibited characteristic Raman peaks at $620.5{\;\mathrm{cm}}^{-1}$, $1197.7{\;\mathrm{cm}}^{-1}$, $1278.6{\;\mathrm{cm}}^{-1}$, $1356.6{\;\mathrm{cm}}^{-1}$, $1502.8{\;\mathrm{cm}}^{-1}$, and $1642.4{\;\mathrm{cm}}^{-1}$, similar to the Raman frequencies of RhB on plasma-synthesized AuNPs. In addition, major Raman peak intensities of RhB on commercial gold nanoparticles were slightly different from those of plasma-synthesized gold nanoparticles. These differences can be ascribed to the different fabrication method of gold nanoparticles and the different shell surrounding the AuNP core. A PEG shell surrounding the AuNP core can affect the binding between AuNPs and RhB molecules. Next, plasma-synthesized AuNPs were tested for the optical sensing of another molecule, methylene blue (MB). For this purpose, we coated MB on the AuNP-paper and measured the Raman spectra. Figure 8D shows the Raman spectra of MB adsorbed on the AuNP-paper. The Raman spectra of MB on AuNPs exhibited characteristic Raman peaks at $448.3{\;\mathrm{cm}}^{-1}$ and $1621.4{\;\mathrm{cm}}^{-1}$, which we attributed to C−N−C skeletal bending and C−C stretching modes [45,46]. This result indicates that plasma-synthesized AuNPs can be used to produce a Raman-sensitive AuNP-paper platform. To summarize, such an AuNP-paper-based Raman sensing platform has several merits: (1) it requires a small volume sample, and is cheap, flexible, and biocompatible; (2) it can discriminate between normal and damaged areas; and, (3) it is easy to load gold nanoparticles and sensing molecules on the paper surface. The spatial resolution and homogeneity of the sensing materials on the AuNP-paper are limited due to the use of filter paper and dip-coating method [47].

## 4. Conclusions

Herein, we described the synthesis of PEG-functionalized gold nanoparticles from tetrachloroauric acid and PEG using an argon plasma jet without additional reducing chemicals. Au@PEG NPs with a mean particle diameter of 32.5 nm were obtained after plasma treatment for 10 min. The presence of PEG in the solution assisted the formation of Au nanoparticle cores. Au@PEG NPs were coated on filter paper by a simple soaking process to produce a Raman-sensitive AuNP-paper platform. The AuNP-paper acted as a surface-enhanced Raman scattering platform when used to analyze the degradation of RhB dye. In summary, this study presents a quick and cost-effective process for the Raman sensing of plasma-synthesized Au@PEG NPs.
